# Bilateral Interference in Motor Performance in Homologous vs. Non-homologous Proximal and Distal Effectors

**DOI:** 10.3389/fpsyg.2021.680268

**Published:** 2021-07-12

**Authors:** Morten Andreas Aune, Håvard Lorås, Alexander Nynes, Tore Kristian Aune

**Affiliations:** ^1^Department of Sport Science, Sport and Human Movement Science Research Group (SaHMS), Nord University, Levanger, Norway; ^2^Department of Teacher Education, Faculty of Social and Educational Sciences, NTNU – Norwegian University of Science and Technology, Trondheim, Norway

**Keywords:** bimanual coordination, interhemispheric communication, movement constraints, bilateral interference, upper-limb coordination

## Abstract

Performance of bimanual motor actions requires coordinated and integrated bilateral communication, but in some bimanual tasks, neural interactions and crosstalk might cause bilateral interference. The level of interference probably depends on the proportions of bilateral interneurons connecting homologous areas of the motor cortex in the two hemispheres. The neuromuscular system for proximal muscles has a higher number of bilateral interneurons connecting homologous areas of the motor cortex compared to distal muscles. Based on the differences in neurophysiological organization for proximal vs. distal effectors in the upper extremities, the purpose of the present experiment was to evaluate how the level of bilateral interference depends on whether the bilateral interference task is performed with homologous or non-homologous effectors as the primary task. Fourteen participants first performed a unilateral primary motor task with the dominant arm with (1) proximal and (2) distal controlled joysticks. Performance in the unilateral condition with the dominant arm was compared to the same effector’s performance when two different bilateral interference tasks were performed simultaneously with the non-dominant arm. The two different bilateral interference tasks were subdivided into (1) homologous and (2) non-homologous effectors. The results showed a significant decrease in performance for both proximal and distal controlled joysticks, and this effect was independent of whether the bilateral interference tasks were introduced with homologous or non-homologous effectors. The overall performance decrease as a result of bilateral interference was larger for proximal compared to distal controlled joysticks. Furthermore, a proximal bilateral interference caused a larger performance decrement independent of whether the primary motor task was controlled by a proximal or distal joystick. A novel finding was that the distal joystick performance equally interfered with either homologous (distal bilateral interference) or non-homologous (proximal bilateral interference) interference tasks performed simultaneously. The results indicate that the proximal–distal distinction is an important organismic constraint on motor control and for understanding bilateral communication and interference in general and, in particular, how bilateral interference caused by homologous vs. non-homologous effectors impacts motor performance for proximal and distal effectors. The results seem to map neuroanatomical and neurophysiological differences for these effectors.

## Introduction

The control and coordination of various bimanual motor actions have interested researchers for decades, and several constraints have been proposed that affect bimanual motor actions (Kelso, 1984; Pashler, 1994; Swinnen, 2002; Wenderoth et al., 2003; Mechsner and Knoblich, 2004; Kolb and Whishaw, 2009; Swinnen and Gooijers, 2015). The predominant theoretical frameworks for understanding bimanual motor actions are from the motoric and neurophysiological approaches (Swinnen, 2002; Wenderoth et al., 2003), and from the perceptual and motor-planning processes (Mechsner and Knoblich, 2004; Swinnen and Gooijers, 2015).

From a motoric and neurophysiological approach, bimanual motor actions require appropriate bilateral communication (Swinnen, 2002; Gooijers and Swinnen, 2014). Normally, the two arms and hands benefit from bilateral communication and neural crosstalk between body sides to achieve a common goal. Neural crosstalk refers to mirrored neural activity sent to homologous contralateral brain areas and muscles during bimanual motor actions (Cattaert et al., 1999; Swinnen, 2002; Kennedy et al., 2014). Some motor actions require independent control and coordination between body sides, and in such bimanual tasks, coordination of inhibitory and excitatory neural crosstalk is detrimental for high performance. Unfortunately, in some bimanual motor actions, neural crosstalk can cause bimanual interference of motor performance (e.g., Aune et al., 2020).

The identification of interference mechanisms between body sides in different bimanual motor tasks is essential for understanding bimanual motor control and coordination in general and, in particular, how bimanual interference between arms and hands illuminates a behavioral constraint for bimanual motor control and coordination. Different bimanual coordination tasks are considered to be an important entry point for research on bimanual interference, particularly when the two arms are operated simultaneously in everyday tasks or in sport-specific tasks. Several studies suggest that control and coordination of bimanual tasks are especially compromised when the arms and hands have to move simultaneously and independently with different spatial and/or temporal trajectories (Sherwood, 1994; Spijkers and Heuer, 1995; Franz et al., 1996; Cattaert et al., 1999; Heuer et al., 2001; Swinnen et al., 2001; Wenderoth et al., 2003; Levin et al., 2004; Aune et al., 2013; Nemani et al., 2018). For example, research has shown that bimanual interference emerges when two limbs must be moved along different trajectories and when the action is conducted under different task parameters (Wenderoth et al., 2003). The main parameters affecting bimanual interference appear to be differences in amplitude and direction of movement, along with velocity and shape assimilation (Franz, 1997; Walter et al., 2001; Weigelt et al., 2007; Calvin et al., 2010), and force modulation (Heuer et al., 2001; Kennedy et al., 2014, 2015). In addition, differences in movement frequency and relative phase relations influence bimanual coordination patterns and performance (Kelso, 1984; Kelso et al., 1986). In general, a high degree of stability in bimanual coordination is associated with symmetrical bimanual movements where both arms are moved along the same trajectory and with a common goal (Swinnen and Wenderoth, 2004) due to the coactivation of homologous areas of the primary and supplementary motor cortex and muscles across body sides (Cohen, 1971; Kelso, 1984; Carson, 1990; Cattaert et al., 1999; Swinnen and Gooijers, 2015). Such types of bimanual movements consist of a high level of similarity in neural activity between the contralateral muscles, and the activation of ipsilateral neural signals is congruent (Kelso, 1984; Marteniuk et al., 1984; Kagerer et al., 2003; Maki et al., 2008). Neural crosstalk connecting homologous muscles causes strong bilateral interaction between these muscles (e.g., Cohen, 1971; Kelso, 1984; Cardoso de Oliveira, 2002; Swinnen, 2002).

Additionally, it is suggested that asymmetrical bimanual motor tasks which require a more differentiated role for each arm interfere more than bimanual movements where each arm is moved along the same trajectory (e.g., Marteniuk et al., 1984; Summers et al., 1993; Franz et al., 1996; Swinnen, 2002). The neural activity in bilateral motor tasks with different task parameters (e.g., trajectories, amplitude, and frequency) can, therefore, cause negative neural crosstalk (in terms of motor performance) between the homologous primary and supplementary motor cortex and muscles, where the neural activity between the limbs is not similar (Marteniuk et al., 1984; Cardoso de Oliveira, 2002). In this type of neural crosstalk, the mirrored neural activity from the contralateral arm conflicts with an appropriate neural activity associated with the specific motor task in the contralateral arm and, thus, causes bilateral interference (Marteniuk et al., 1984; Summers et al., 1993; Cardoso de Oliveira, 2002; Kagerer et al., 2003). Therefore, because of a stronger bilateral interaction and crosstalk between homologous compared to non-homologous brain areas and muscles, it can be expected that the potential bilateral interference is higher for homologous muscle groups compared to non-homologous muscle groups. Respectively, as observed by Kennedy et al. (2014), in bimanual force control, an increase or decrease in force production by one limb can lead to a corresponding change in the force production in homologous muscles in the contralateral arm.

However, some studies have shown that stable bimanual movements could be associated with non-homologous muscle groups, for example, during multi-joint movements (e.g., Kelso et al., 1991; Buchanan and Kelso, 1993), when bimanual in-phase movements were manipulated with visual feedback to create perceptual symmetry (e.g., Mechsner et al., 2001; Mechsner and Knoblich, 2004) or with the use of non-homologous limbs in iso-directional movements (Serrien and Swinnen, 1997; Serrien et al., 2001). Thus, the bilateral communication and bilateral interference are probably highly task specific.

To understand the effect of bilateral interference in general and between homologous and non-homologous muscles, knowledge is necessary about the bilateral organization of the neuromuscular system and how neural crosstalk is a crucial, organismic constraint in bilateral communication for the control and coordination of different motor tasks and different effectors. From a neurophysiological perspective, the corpus callosum and interneurons in the spinal cord play a prominent role in mediated bilateral communication required for the execution of bimanual tasks (Jeeves et al., 1988; Hellige, 1993; Heuer et al., 1995; Swinnen, 2002; Wenderoth et al., 2003; Cohen, 1971; Swinnen and Gooijers, 2015). Bilateral neural interactions are essential for the transfer and integration of information between cortical areas in the hemispheres and bilateral interactions in the corticospinal tracts. Neural crosstalk between hemispheres can be inhibitory and decrease neural drive to the contralateral muscles during some bilateral motor actions (Delwaide and Pepin, 1991; Ferbert et al., 1992; Bannatyne et al., 2003; Hübers et al., 2008; Takeuchi et al., 2012), but in other tasks, the neural crosstalk between hemispheres can be inhibitory and increase neural drive to the contralateral muscles during bilateral motor actions (Kinsbourne, 1975; Serrien and Brown, 2002; Hortobágyi et al., 2003; Bloom and Hynd, 2005; Jankowska et al., 2005a,b; Bannatyne et al., 2006; Liuzzi et al., 2011). Thus, whether the neural drive to the contralateral hemisphere and muscles increases or decreases in bimanual motor actions and muscle contractions depends on the type of movement and purpose of the motor actions (Oda and Moritani, 1995; Taniguchi et al., 2001; Schultze et al., 2002; Khodiguian et al., 2003).

However, it would be interesting to study differences in bilateral interference associated with specific homologous and non-homologous effectors and whether it differs for proximal and distal muscles. The numbers of transcallosal projections (commissural fibers through corpus callosum) and commissural interneurons in the spinal cord connecting proximal muscles are higher compared to distal muscles in primates (Pandya and Vignolo, 1971; Jenny, 1979; Gould et al., 1986; Rouiller et al., 1994; Brodal, 2004; Jankowska et al., 2005b; Pierrot-Deseilligny and Burke, 2005), and they probably increase the potential for bilateral interference for proximal effectors (Aune et al., 2020).

It should also be noted that distal arm muscles are mainly innervated by monosynaptic connections through the lateral corticospinal tract, while proximal arm muscles are mainly innervated through polysynaptic connections in the ventromedial corticospinal tract (Kuypers, 1978; Palmer and Ashby, 1992; Brodal, 2004). Thus, it is suggested that, as a consequence of the greater proportion of monosynaptic connections between the motor cortex and distal muscles, the potential for bilateral interference and neural leakage for those muscles might be weakened (Aune et al., 2020). Such neuroanatomical and neurophysiological differences between the proximal and distal muscles impact the potential for bilateral communication and bilateral interference for proximal and distal effectors, which in some tasks might be an advantage, while in others it might be a disadvantage.

In a previous study, we observed a larger bimanual interference for proximal effectors compared to distal effectors (Aune et al., 2020) in simultaneous non-isomorphic movements of the arms. A limitation of that study is that it did not restrict the interference task to activate specific homologous or non-homologous effectors, nor did it manage to determine whether the bilateral interference was influenced by homologous or non-homologous effectors.

Previously research comparing bilateral interference between homologous and non-homologous muscles has extensively focused on bimanual finger and hand dexterities that require bimanual force control (e.g., Kennedy et al., 2014, 2015) or bimanual coordination with different relative phase or amplitude requirements (e.g., Cohen, 1971; Kelso, 1984). In addition, some studies of bimanual movements have analyzed the effect of perceptual constraints (visual feedback) on bimanual coordination (Mechsner et al., 2001; Mechsner and Knoblich, 2004). A combination of the different parameters used in the respective studies demonstrate the complexity of bimanual motor control and coordination and make it difficult to compare different studies to understand the constraints affecting bimanual interactions.

However, such types of bimanual tasks are more distant from everyday tasks, and therefore, it would be interesting to study bimanual coordination from a more practical approach. In addition, another constraint for motor control and coordination in upper extremities that it is necessary to be aware for understanding the distinction between proximal and distal effectors is their differences in biomechanics. Proximal and distal effectors have different lengths of segments and eigen frequencies, and to compare and understand the proximal and distal differences in motor control, experimental tasks have to be normalized as much as possible.

Based on the presented considerations, the specific aim of the current study was to investigate how a joystick controlled by proximal or distal effectors (a primary task) performed with the dominant arm is interfered with by the introduction of a bilateral interference task performed with the non-dominant arm with either homologous or non-homologous effectors. It was hypothesized that bimanual motor actions, performed with bilateral homologous effectors (distal–distal and proximal–proximal) with different movement trajectories, introduce more bilateral interference for the primary motor task compared to bilateral interference from non-homologous effectors (distal–proximal and proximal–distal) for both proximal and distal effectors. It was further hypothesized that there is a more pronounced bilateral interference in homologous proximal effectors compared to homologous distal effectors.

## Materials and Methods

### Participants

A sample of 14 neurologically healthy university students, seven women (mean age 23.2, *SD* = 6.5 years) and seven men (mean age 25.1, *SD* = 1.5 years) were recruited and gave informed consent before participating in the study. Based on results from the previous studies with the same experimental task (Aune et al., 2017, 2020), it was estimated that this sample size was sufficient to achieve a power of 80%, a level of significance of 5% (two-sided), and an effect size (partial eta squared) of 0.7 for detecting a main effect of proximal vs. distal effectors on task performance, absolute spatial error (ASE; see data analysis section) with repeated measures ANOVA. All participants were right-handed as indicated by the Edinburgh Handedness Inventory (Oldfield, 1971) with a mean laterality index score of 0.94 (*SD* = 0.06). None of the participants reported any specialized training/practice of the upper extremities. All participants gave informed consent prior to the experimental procedure. The study protocol was evaluated and approved by the Regional Committee for Medical and Health Research Ethics and performed in accordance with the Declaration of Helsinki.

### Primary Motor Task

**Figure 1:**
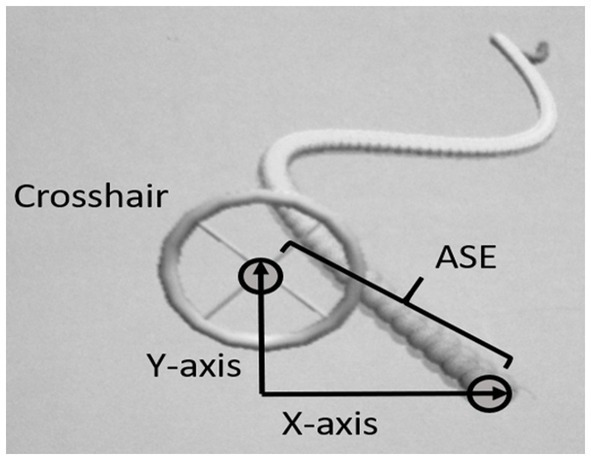
Design of the “moving snake” and calculation of absolute spatial error (ASE).

The motor task used in the present study has been described extensively in the previous studies (Aune et al., 2017, 2020). Briefly, participants were positioned in a custom-made chair 3 m from a screen (148 × 110 cm) and had to use a controllable crosshair to track the head of a 2D virtual “moving snake” as precisely as possible (see Figure 1). The movements consist of a complex two-dimensional (*x* and *y*) periodic waveform made by the head of the snake (the same in every trial). When the center of the crosshair was perfectly positioned on the snake’s head, the color of the head changed, providing instantaneous feedback to the participant. The sampling frequency used for the task was 100 Hz, and each sampling point stored *x* and *y* coordinates for the target and crosshair. The moving snake task was designed using the Unity3D game engine and programmed using C#.

**Figure 2:**
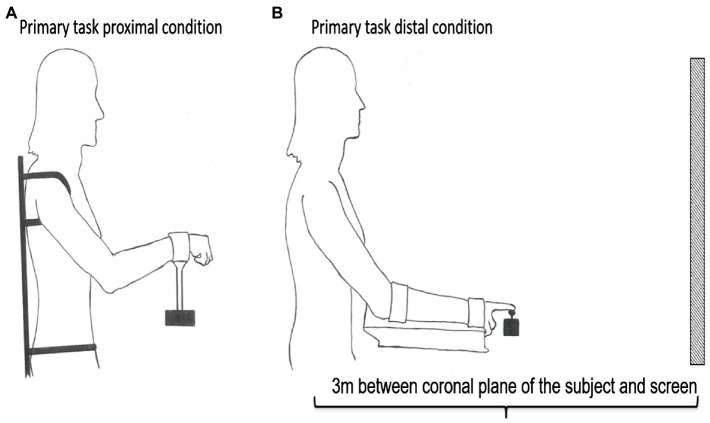
Illustration of the experimental setup of the primary task controlled with either a **(A)** proximal joystick or a **(B)** distal joystick. The subject was positioned seating 3 m from the screen in both conditions. In order to prevent mechanical, postural, and synergist muscle contributions in the proximal **(A)** and distal **(B)** conditions, the participants’ body positions were constrained by clamps and straps as illustrated. The starting position in the proximal condition was calibrated to 45 between the trunk and overarm (humerus) and 130 between humerus and radius **(A)**. The starting position in the distal condition was calibrated to 25 between the trunk and overarm, with the underarm resting in a horizontal position **(B)**.

The crosshair was controlled by two different customized joysticks to perform isolated unilateral movements of the proximal and distal effectors: (1) a customized proximal joystick controlled by the shoulder and elbow and (2) a customized distal joystick controlled by the wrist and index finger (see Figure 2). Both joysticks were operated with the dominant hand. The movement of both the proximal and distal joysticks was a combination of flexion-extension and adduction-abduction and had a range of movement (ROM) set to 30° in each direction from the neutral starting position of the two joysticks. This was done in order to normalize angular ROM and reduce biomechanical differences for the proximal and distal joysticks despite different lengths of segments and eigen frequencies. The custom-made chair and apparatus were used to prevent postural instability, thereby limiting activation to the shoulder–elbow in the proximal condition and the wrist–index finger in the distal condition.

### Bilateral Interference Tasks

**Figure 3:**
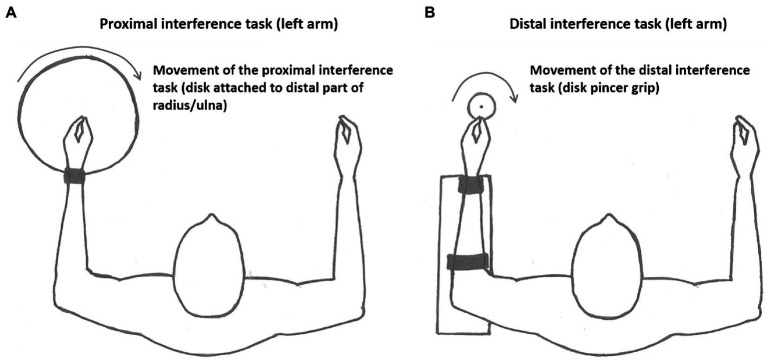
Illustration of the experimental setup of the **(A)** proximal interference task and the **(B)** distal interference task performed with the non-dominant arm. The proximal and distal interference tasks were performed in simultaneously with the primary task in the four different condition: (1) proximal primary task simultaneously with proximal interference task (homologous effectors), (2) proximal primary task simultaneously with distal interference task (non-homologous effectors), (3) distal primary task simultaneously with distal interference task (homologous effectors), and (4) distal primary task simultaneously with proximal interference task (non-homologous effectors).

The proximal and distal bilateral interference tasks (see Figure 3) were performed simultaneously as the primary task with a different movement trajectory compared to the primary task. The interference tasks involved a constrained circular motion performed with the non-dominant arm that consisted of rotating a disk that required either activation of (1) proximal (shoulder and elbow) or (2) distal (wrist and fingers) effectors (see Figure 3). In both bimanual interference tasks, participants were instructed to rotate the disk with an inward rotation direction (clockwise for right-handed participants) continuously at a steady speed of about 1 Hz. For both the proximal and distal interference task, the ROM was set to be approximately the same ROM as the primary task.

### Proximal Interference Task

In the proximal bilateral interference task, participants were restricted to moving a rotating disk (diameter = 30 cm) with their non-dominant shoulder and elbow. The ROM of the proximal interference task was calibrated to correspond to the ROM for the proximal joystick (primary task; see Figure 3).

### Distal Interference Task

In the distal bilateral interference task, participants held a rotating disk (diameter = 5.5 cm) by gripping a small bar that was attached to the disk with their non-dominant index finger and thumb (pincer grip). The ROM of the distal interference task was calibrated to correspond to the ROM for the distal joystick (primary task; see Figure 3).

### Procedure

The experiment required two consecutive days (see also Aune et al., 2017, 2020). On the first day, participants were informed about the task, given a short demonstration, and allowed to familiarize themselves with the experimental setup and the two different joysticks. Subsequently, they completed 20 practice trials with both the proximal and distal joysticks subdivided into four blocks of five trials. Each block was followed up by a 2-min rest period. On the second day, participants completed six different conditions: proximal joystick without interference, with proximal interference, and with distal interference; distal joystick without interference, with proximal interference, and with distal interference. The order in which the proximal- vs. distal-controlled joysticks were tested was counterbalanced across participants, and one practice trial was performed before testing in the different conditions. Three trials were performed in each of the six conditions.

### Data Analysis

In accordance with the previous studies (Aune et al., 2017, 2020), performance in the tracking task was measured as the ASE in positioning of the crosshair relative to the target, measured as the distance between the head of the snake and the middle of the crosshair. The unit of measurement was virtual meters (VM) as defined in the customized software. Absolute spatial error was measured as the distance between the head of the snake and the middle of the crosshair, calculated using Pythagoras equation (see Figure 1):

$$\mathrm{Absolute Spatial Error}\;\left(\mathrm{ASE}\right)=\sqrt{\left(x^{2}+y^{2}\right)}$$

Performance in all conditions was tested over a 50-s epoch, and three trials were performed in each condition. Average ASE was calculated across the three trials and used in subsequent analyses. The bimanual interference task was analyzed by video to confirm that the participants were moving in accordance with the instructions. Trials that temporally had a mean deviation of more than 0.1 Hz were eliminated from further analyses (in total < 5%). The experimental data were processed and administered using a custom-made software using the Microsoft Excel (Version 2012).

### Statistical Analysis

Shapiro–Wilk tests, inspection of Q-Q plots and histograms indicated that all variables were normally distributed. Thus, the effect of bimanual interference on control of the proximal and distal joysticks was examined with a two effector (proximal or distal) × three conditions (no interference, homologous interference, or non-homologous interference) within-subject repeated measures ANOVA on the ASE. In the rm. ANOVA, partial eta squared ($\eta_{p}^{2}$) was applied as the indicator of the effect size interpreted as small effect: 0.01, medium effect: 0.06, and large effect: 0.14 (Cohen, 1988; Richardson, 2011). Post-hoc Bonferroni corrected pairwise comparisons at the level of simple main effects on accuracy (ASE) was conducted with paired samples *t*-tests: proximal effector – no interference vs. proximal or distal interference and distal effector – no interference vs. proximal or distal interference. For dependent *t*-tests, Cohen’s *d_Z_* was applied as a measure of the effect size (Lakens, 2013), in which 0.2, 0.5, and 0.8 were considered small, moderate, and large, respectively (Cohen, 1988). Calculations of 90% CI for partial eta squared were conducted by syntax designed by Professor Karl Wuensch.^1^ All statistical calculations were performed with the Predictive Analytics Software (PASW, IBM, United States; previously SPSS) Version 26.0 with alpha = 0.05 as the criterion for statistical significance.

## Results

**Figure 4:**
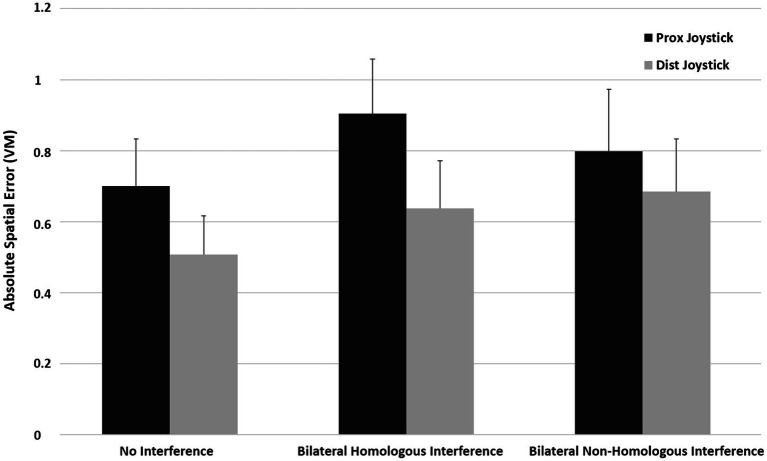
Mean ASE for the proximal- and distal-controlled joystick under conditions of no interference, homologous interference, and non-homologous interference. The error bars illustrate *SD*.

As depicted in Figure 4, the ASE is higher for proximal joystick control compared to distal joystick control in all conditions. Figure 4 also shows that interference increased the ASE in both proximal and distal effectors. A repeated measures (rm) ANOVA indicated a significant effector (proximal or distal) x condition (no interference, homologous interference, or non-homologous interference) interaction effect on ASE [*F*(1, 13) = 10.47, *p <* 0.001, $\eta_{p}^{2}$ = 0.45 (90% CI [0.09, 0.64])]. Further, rm. ANOVA indicated a significant main effect of the proximal vs. distal effector on ASE [*F*(1, 13) = 88.13, *p* < 0.001, $\eta_{p}^{2}$ = 0.87 (90% CI [0.70, 0.92])] with a mean difference of 0.19 virtual meters (95% CI [0.14, 0.24]) and a significant main effect of condition (no interference, homologous interference, or non-homologous interference) on ASE [*F*(1, 13) = 29.82, *p* < 0.001, $\eta_{p}^{2}$ = 0.70 (90% CI [0.37, 0.80])]. Post-hoc analysis indicated significantly higher ASE with homologous interference compared to no interference (mean difference = 0.17, 95% CI [0.1, 0.23], *p* > 0.001), significantly higher ASE with non-homologous interference compared to no interference (mean difference = 0.14, 95% CI [0.06, 0.20], *p* < 0.001), and no significant difference in ASE with homologous interference compared to non-homologous interference (mean difference = 0.03, 95% CI [−0.02, 0.07], *p* = 0.279).

### Proximal Effector: Homologous vs. Non-homologous Interference

Further post-hoc analysis with paired samples *t*-tests indicated significantly higher ASE at the level of the proximal effector (mean difference = 0.21, 95% CI [0.14, 0.27]) with homologous interference compared to no interference [*t*(13) = 7.02, *p* < 0.001, *d_Z_* = 1.87 (95% CI [0.98, 2.75])], significantly higher ASE (mean difference = 0.09, 95% CI [0.01, 0.18]) with non-homologous interference compared to no interference [*t*(13) = 2.49, *p* < 0.027, *d_Z_* = 0.67 (95% CI [0.08, 1.24])], and significantly higher ASE (mean difference = 0.11, 95% CI [0.06, 0.16]) with homologous interference compared to non-homologous interference [*t*(13) = 4.49, *p* = 0.001, *d_Z_* = 1.20 (95% CI [0.49, 1.88]); see Figure 4].

### Distal Effector: Homologous vs. Non-homologous Interference

Similarly, at the level of the distal effector (see Figure 4), ASE was significantly higher (mean difference = 0.18, 95% CI [0.13, 0.23]) with non-homologous interference compared to no interference [*t*(13) = 7.46, *p* < 0.001, *d_Z_* = 1.99 (95% CI [1.06, 2.90])] and significantly higher (mean difference = 0.13, 95% CI [0.07, 0.19]) with homologous interference compared to no interference [*t*(13) = 4.43, *p* = 0.001, *d_Z_* = 1.18 (95% CI [0.48, 1.86])]. No significant difference in ASE (mean difference = 0.05, 95% CI [−0.003, 0.10]), however, was found between non-homologous interference compared to homologous interference [*t*(13) = 2.02, *p* = 0.065, *d_Z_* = 0.54 (95% CI [−0.03, 1.09])].

## Discussion

In a recent experiment, we found a more pronounced bilateral interference in proximal effectors compared to distal effectors, but unfortunately, the experiment did not discriminate between whether the bilateral interference was influenced by homologous or non-homologous bimanual actions (Aune et al., 2020). Consequently, the purpose of the current study was to examine potential differences in bilateral interference between homologous interference vs. non-homologous interference tasks in proximal and distal effectors of the upper extremities. To compare the bilateral interference on motor performance (increased ASE in the primary task) in proximal and distal effectors, a unilateral motor task (primary task only) was compared to a bimanual motor task (with the primary task and bilateral interference task performed simultaneously).

The general results of the present study are in congruence with our earlier studies using joystick control as the primary experimental task. The joystick control was operated more precisely with distal effectors compared to proximal effectors, and in addition, a general bilateral interference (a decrease in spatial accuracy for the primary task in the bimanual condition) was observed for both the proximal- and distal-controlled joysticks with either a homologous or a non-homologous interference task (for comparable findings, see Aune et al., 2017, 2020). The present results are in accordance with the literature describing the advantage of using distal effectors (small musculature) for accuracy in fine motor movements that requires a high degree of accuracy, while proximal effectors (large musculature) are more adequate for gross movements that require less accuracy (Bernstein, 2014; Magill and Anderson, 2017).

Bimanual coordination strongly depends on a complex pattern of neural activity and continuous bilateral communication between body sides to perform well-coordinated bimanual movements (e.g., Serrien and Brown, 2002; Swinnen, 2002; Swinnen and Wenderoth, 2004). These results show that asymmetrical bimanual movements with different task goals for each limb cause bilateral neural crosstalk that is negative for motor control and coordination independent of whether the movements are between homologous or non-homologous muscles (Marteniuk et al., 1984; Cardoso de Oliveira, 2002), and subsequently, bimanual coordination and bilateral neural crosstalk generate bilateral neural signals that are consequential for optimal motor control of both proximal and distal joysticks (e.g., Kelso, 1984; Franz et al., 1991; Swinnen et al., 1991; Cardoso de Oliveira, 2002; Kagerer et al., 2003; Aune et al., 2020).

A specific aim of the current study was to investigate whether the introduction of a bimanual motor action with homologous or non-homologous effectors performed with the non-dominant arm interferes with the joystick controlled by the dominant arm’s proximal or distal effectors (the primary task).

The results confirmed the hypothesis of a more pronounced bilateral interference for the primary motor task (distal–distal and proximal–proximal) in bimanual motor actions performed with bilateral homologous effectors compared to bilateral interference from non-homologous (distal–proximal and proximal–distal) effectors for both proximal and distal effectors.

### Homologous Bilateral Interference in Proximal and Distal Effectors

A detailed analysis of the current results confirmed the hypothesis of a more pronounced bilateral interference between homologous proximal effectors (proximal joystick with proximal interference task) compared to homologous distal effectors (distal joystick with distal interference task). As an explanation of these findings, neural crosstalk has been suggested to be a crucial organismic constraint in bimanual coordination (e.g., Serrien and Brown, 2002; Swinnen, 2002; Swinnen and Wenderoth, 2004; Carson, 2005). Also, in bimanual movements with different trajectories for each limb, neural crosstalk caused a mirrored neural activity to the contralateral arm. This conflicts with an appropriate neural activity to the specific motor task in the contralateral arm (Marteniuk et al., 1984; Summers et al., 1993; Cardoso de Oliveira, 2002; Kagerer et al., 2003). The more-pronounced bilateral homologous interference to proximal effectors found in the current study might, therefore, be explained by a higher potential of bilateral communication to proximal muscles than to distal muscles (Brinkman and Kuypers, 1972; Jankowska, 1992; Palmer and Ashby, 1992). A higher number of commissural fibers through the corpus callosum and commissural interneurons in the spinal cord would increase the potential of neural crosstalk and bilateral interference in proximal effectors (Aune et al., 2020). Hence, homologous proximal effectors have a higher potential to bilaterally interact with contralateral motor neurons and generate a neural activity that is not congruent with the motor performance in the proximal effector and thus causes bilateral interference (Marteniuk et al., 1984; Cardoso de Oliveira, 2002; Kagerer et al., 2003; Aune et al., 2020). These results align with those of earlier findings that have described a more pronounced bilateral interference in proximal than in distal effectors. These results describe a proximal–distal gradient in bilateral interference in homologous proximal and distal effectors that is assumed to be mediated by differences in the potential for bilateral communication to proximal and distal muscles (Aune et al., 2020). Based on these considerations, the motor control of a distal joystick might have a greater potential to maintain stability in movement patterns even though it is interfered with by a bilateral interference task in the non-dominant arm, because distal effectors are more precise in fine motor skills proximal effectors (Bernstein, 2014; Magill and Anderson, 2017).

### Non-homologous Bilateral Interference in Proximal and Distal Effectors

The analysis of the impact of bilateral interference from non-homologous effectors for proximal and distal joysticks generated several novel findings. For the proximal-controlled joystick, the hypothesized greater bilateral interference from homologous compared to non-homologous effectors on the interference task was confirmed. These results are associated with and seem to represent the expected neuroanatomical and neurophysiological differences between proximal and distal muscles. As described above, the proximal muscles have a high number of commissural fibers through the corpus callosum between homologous proximal muscles, and it is the neuroanatomical origin of the high level of interhemispheric communication and bilateral transfer of information between proximal homologous cortical areas in the supplementary and primary motor cortex. The distal interference task (non-homologous interference) interferes significantly less with the proximal joystick (primary task). This might be because of a lower potential of bilateral communication, and accordingly a relatively low level of neural crosstalk between the body sides in the current condition (Aune et al., 2020).

In contrast, the performance with the distal joystick as a primary task was equally interfered with by both the homologous effector (distal bilateral interference task) and the non-homologous effector (proximal bilateral interference task) performed simultaneously in the current study. These findings can be explained by the lower potential for bilateral communication and neural crosstalk between distal muscles at both the cortical and spinal level (e.g., Brinkman and Kuypers, 1972; Palmer and Ashby, 1992). As designated, compared to the proximal muscles, the distal muscles have no or few commissural fibers through the corpus callosum between homologous distal muscle cortical areas in the supplementary and primary motor cortex, and the neuroanatomical design constraining bilateral interference through interhemispheric crosstalk between distal effectors. The equivalent bilateral interference from the homologous and non-homologous effectors for the distal joystick might be related to intrahemispheric crosstalk between proximal and distal somatotopic areas. Subsequently, proximal and distal muscles, to some extent, share neurophysiological resources and ipsilateral brain areas in the execution of motor tasks (Brodal, 2004). Accordingly, the proximal interference task has a greater potential to interfere with a bilateral non-homologous primary task (in the present experiment, the control of the distal joystick) as a result of both inter- and intrahemispheric neural crosstalk. In the distal condition (control of the distal joystick as the primary task), the non-homologous proximal interference task in addition to interhemispheric neural crosstalk might cause additional interference through intrahemispheric neural crosstalk between proximal and distal ipsilateral somatotopic brain areas that interfere with the execution of motor control and performance of the distal joystick. However, attentional factors associated with the task environment might also influence bilateral interference (Mechsner and Knoblich, 2004; Swinnen and Gooijers, 2015). Attentional factors, such as the vision of the proximal interference task, probably require more perceptual attention, and subsequently, it might interfere to a greater extent with perceptual and motor planning processes.

### Limitations of the Study and Future Perspectives

The functional and behavioral data collected in the current study do not measure bilateral interference directly, and measures of brain activity could provide additional and more-detailed insights into the bilateral interference of proximal and distal muscles.

In future research, it would be interesting to include measures of both brain activity, for example, electroencephalogram (EEG) or functional magnetic resonance imaging, and muscle activity through, for example, electromyography (EMG), to provide additional understanding of how inhibitory and excitatory interactions cause bilateral interference. A direct measure of neural activation by EEG and EMG measurements can also indicate whether a bilateral interference is caused by cortical interactions alone, or whether spinal interactions should be considered in addition. A recent study demonstrated bilateral EMG coherence of neural crosstalk between triceps muscles and showed that it can stabilize 1:1 in-phase bimanual coordination patterns when contralateral and ipsilateral neural signals are congruent between two effectors (Wang et al., 2021). Based on that, it would be interesting in the future studies to evaluate potential differences in bilateral EMG coherence between proximal and distal muscles. In addition, it would be interesting to analyze more variables and aspects that can describe and explain differences in bilateral interference between proximal and distal muscles in both the dominant and the non-dominant arm, for example, bimanual phase relations, distortions in trajectory trace, cycling frequency, and jerk. In addition, it has been shown that force modulation is a specific task parameter that influences bilateral interference between homologues and non-homologues muscles (Kennedy et al., 2014, 2015). Therefore, it would be interesting in the future research to manipulate and adjust the resistance load in the proximal and distal joysticks to evaluate how force influences movement accuracy.

Another perspective that needs consideration is that people are usually more accustomed to using their fingers and wrists to control joysticks rather than using the shoulder and elbow. Thus, the latter might require a higher cognitive load (e.g., motor planning) that could possibly cause more bilateral interference from proximal effector tasks (Aune et al., 2020). For that reason, it would be possible to evaluate potential differences in bilateral interference in proximal effectors and distal effectors after practicing bimanual coordination tasks. Furthermore, some research studies have shown that neural crosstalk is asymmetric in nature where the dominant limb leads to a greater bilateral interference in the non-dominant arm compared to bilateral interference from the non-dominant arm to the dominant arm (e.g., Cattaert et al., 1999; Kagerer et al., 2003; de Poel et al., 2007; Maki et al., 2008; Kennedy et al., 2017; Panzer et al., 2021). Therefore, it would be interesting to study bilateral interference asymmetry with proximal effectors and distal effectors.

### Practical Implications

Increased knowledge of the proximal–distal distinction as an important organismic constraint on bimanual motor control and coordination, and understanding bilateral communication and how interference affects motor performance have practical implications for many bimanual actions performed in daily life. The experiment conducted for this study discriminated between the effect of homologous vs. non-homologous bilateral interference, and the results demonstrated differences in the impact of bilateral interference on motor performance for proximal-controlled joysticks compared to distal-controlled joysticks. This knowledge is important when optimizing human–machine interfaces, such as designing different steering devices for controlling cars, aircraft, drones, and such. Based on these findings, a distal-controlled joystick is likely to be recommended for optimizing, for example, human–machine interfaces to prevent bilateral interference.

## Conclusion

The purpose of the experiment conducted for the present study was to evaluate how the level of bilateral interference depends on whether the bilateral interference task is performed with homologous or non-homologous effectors as the primary task, specifically, the impact on the performance of proximal and distal joysticks. The experiment managed to discriminate the effect of homologous vs. non-homologous bilateral interference, and the results showed a significant decrease in performance for both proximal- and distal-controlled joysticks as an effect independent of whether the bilateral interference tasks were introduced with either homologous or non-homologous effectors. More specifically, the overall performance decrease caused by bilateral interference was larger for the proximal-controlled joystick compared to the distal-controlled joystick, and in addition, a proximal bilateral interference caused a larger performance reduction independent whether the primary motor task was controlled by a proximal or distal joystick. Most interestingly, the distal joystick performance was interfered with equally by homologous (distal bilateral interference task) and non-homologous (proximal bilateral interference task) tasks performed simultaneously. The results demonstrate that the proximal-distal distinction is an important organismic constraint on motor control and for the understanding of bilateral communication and interference in general and, in particular, how bilateral interference caused by homologous vs. non-homologous impacts motor performance for proximal and distal effectors. The present results appear to map neuroanatomical and neurophysiological differences between proximal effectors and distal effectors and increase the understanding of bilateral communication and interference in general and, in particular, how bilateral interference caused by homologous vs. non-homologous impacts motor performance for proximal and distal effectors in the upper extremities.

## Data Availability Statement

The raw data supporting the conclusions of this article will be made available by the authors, without undue reservation.

## Ethics Statement

The studies involving human participants were reviewed and approved by the Regional Committee for Medical and Health Research Ethics. The patients/participants provided their written informed consent to participate in this study.

## Author Contributions

All authors listed have made a substantial, direct and intellectual contribution to the work, and approved it for publication. All authors made contribution to the conception and design of the study, acquisition of the data, analysis and interpretation of the data, drafting the manuscript, revising the manuscript for important intellectual content, and approval of the final version of the manuscript to be published.

### Conflict of Interest

The authors declare that the research was conducted in the absence of any commercial or financial relationships that could be construed as a potential conflict of interest.

## Footnotes

^1^http://core.ecu.edu/psyc/wuenschk/SPSS/SPSS-Programs.htm
